# An FPGA-Based SiNW-FET Biosensing System for Real-Time Viral Detection: Hardware Amplification and 1D CNN for Adaptive Noise Reduction

**DOI:** 10.3390/s25010236

**Published:** 2025-01-03

**Authors:** Ahmed Hadded, Mossaad Ben Ayed, Shaya A. Alshaya

**Affiliations:** 1 Computer and Embedded System Laboratory, National Engineering School of Sfax, Sfax University, Sfax 3038, Tunisia; ahmed.hadded@enis.tn; 2 Electronic Industrial, ENISo, Sousse University, Sousse 4054, Tunisia; 3 Department of Computer Science, Faculty of Sciences and Humanities Sciences, Majmaah University, Al Majmaah 11952, Saudi Arabia

**Keywords:** SiNW-FET biosensor, impedance-based sensing, FPGA-based 1D CNN, noise reduction in biosensing, real-time viral detection

## Abstract

Impedance-based biosensing has emerged as a critical technology for high-sensitivity biomolecular detection, yet traditional approaches often rely on bulky, costly impedance analyzers, limiting their portability and usability in point-of-care applications. Addressing these limitations, this paper proposes an advanced biosensing system integrating a Silicon Nanowire Field-Effect Transistor (SiNW-FET) biosensor with a high-gain amplification circuit and a 1D Convolutional Neural Network (CNN) implemented on FPGA hardware. This attempt combines SiNW-FET biosensing technology with FPGA-implemented deep learning noise reduction, creating a compact system capable of real-time viral detection with minimal computational latency. The integration of a 1D CNN model on FPGA hardware for adaptive, non-linear noise filtering sets this design apart from conventional filtering approaches by achieving high accuracy and low power consumption in a portable format. This integration of SiNW-FET with FPGA-based CNN noise reduction offers a unique approach, as prior noise reduction techniques for biosensors typically rely on linear filtering or digital smoothing, which lack adaptive capabilities for complex, non-linear noise patterns. By introducing the 1D CNN on FPGA, this architecture enables real-time, high-fidelity noise reduction, preserving critical signal characteristics without compromising processing speed. Notably, the findings presented in this work are based exclusively on comprehensive simulations using COMSOL and MATLAB, as no physical prototypes or biomarker detection experiments were conducted. The SiNW-FET biosensor, functionalized with antibodies specific to viral antigens, detects impedance shifts caused by antibody–antigen interactions, providing a highly sensitive platform for viral detection. A high-gain folded-cascade amplifier enhances the Signal-to-Noise Ratio (SNR) to approximately 70 dB, verified through COMSOL and MATLAB simulations. Additionally, a 1D CNN model is employed for adaptive noise reduction, filtering out non-linear noise patterns and achieving an approximate 75% noise reduction across a broad frequency range. The CNN model, implemented on an Altera DE2 FPGA, enables high-throughput, low-latency signal processing, making the system viable for real-time applications. Performance evaluations confirmed the proposed system’s capability to enhance the SNR significantly while maintaining a compact and energy-efficient design suitable for portable diagnostics. This integrated architecture thus provides a powerful solution for high-precision, real-time viral detection, and continuous health monitoring, advancing the role of biosensors in accessible point-of-care diagnostics.

## 1. Introduction

The increasing demand for smart sensor systems capable of detecting airborne viruses has intensified the development of advanced biosensing technologies [1,2,3]. Such systems are essential for rapid, accurate detection of pathogens, especially in applications where timely information is critical to public health and safety. Among the various types of biosensors, selecting a sensor with high sensitivity and reliability is crucial for ensuring accurate detection. Traditional sensors, including optical and electrochemical biosensors, have demonstrated efficacy in molecular detection; however, their limitations in terms of response time, sensitivity, and adaptability to low-concentration targets make them less suitable for detecting airborne viruses in dynamic environments [4,5].

Silicon Nanowire Field-Effect Transistor (SiNW-FET) biosensors have emerged as a highly effective solution for airborne virus detection [6,7]. Their nanoscale sensitivity, selectivity, and capacity to detect minute biomolecular interactions position SiNW-FET biosensors as optimal candidates for this application. By functionalizing silicon nanowires with antibody receptors tailored to bind specific viral antigens, SiNW-FET biosensors can translate molecular interactions into measurable electrical signals. However, these signals are often weak, necessitating amplification for accurate interpretation [7,8]. This introduces a major challenge: ensuring a high Signal-to-Noise Ratio (SNR) to enhance detection precision without compromising the sensor’s real-time response capabilities. Traditional noise reduction techniques, such as linear amplification and filtering, are often insufficient to address non-linear and time-varying noise profiles characteristic of biosensor data, which may obscure important signal features.

To address these challenges, this paper proposes an integrated approach combining hardware-based preamplification with machine learning. High-gain folded-cascade preamplifiers are employed to increase the SNR, while a 1D Convolutional Neural Network (CNN) model further refines the signal by adaptively filtering complex noise patterns, effectively overcoming the limitations of traditional linear noise reduction methods [9]. The proposed CNN model, designed for time-series data processing, offers a dynamic approach to noise reduction, preserving critical signal features essential for accurate viral detection.

Real-time detection introduces further complexity, as the computational demands of AI-driven noise reduction models can hinder prompt data processing [10]. To meet the need for real-time operation without compromising on processing power, this work implements the CNN model as a hardware accelerator on an FPGA, leveraging parallel processing capabilities to achieve low-latency, high-throughput noise reduction.

Unlike traditional methods, which rely on static filters, this study leverages a 1D CNN model deployed on FPGA to dynamically learn and filter complex noise patterns in real time. This hybrid system, uniting SiNW-FET biosensors with FPGA-based AI, marks a pioneering approach to applying deep learning to enhance signal fidelity in point-of-care biosensing applications.

In light of this brief introduction, this paper presents a SiNW-FET-based sensor system enhanced by hardware amplification and FPGA-accelerated 1D CNN noise reduction, offering a robust, real-time solution for airborne virus detection with high precision and reliability. It is essential to note that the findings in this study are derived entirely from comprehensive simulations conducted using COMSOL and MATLAB. No physical device prototypes or biomarker detection experiments were performed. The simulated results provide a robust foundation for evaluating the system’s design and functionality under controlled conditions, offering insights that can inform future experimental validation and practical implementation.

The remainder of this paper is organized as follows. Section 2 reviews related work in SiNW-FET biosensing and noise reduction techniques. Section 3 provides the system design and specifications, covering the biosensor architecture, amplification circuit, and the proposed 1D CNN model for noise reduction. Section 4 details the implementation of the CNN on FPGA hardware and discusses the real-time processing capabilities of the proposed system. In Section 5, performance evaluation results, including the Signal-to-Noise Ratio (SNR), latency, resource utilization, and power efficiency, are presented. Finally, Section 6 concludes the paper with a summary of findings and discusses future research directions for enhancing real-time biosensing applications.

## 2. Related Works

Recent advances in biosensing technologies have driven significant research into improving the sensitivity, specificity, and robustness of sensors for detecting biomolecular interactions, particularly in medical and environmental applications. Silicon Nanowire Field-Effect Transistor (SiNW-FET) biosensors, in particular, have garnered attention due to their nanoscale sensitivity, enabling the detection of viral pathogens and other biomolecules at minimal concentrations. Previous works have primarily focused on enhancing SiNW-FET sensor designs and signal processing techniques to overcome noise interference and improve detection accuracy [7,11].

In traditional SiNW-FET biosensor research, one commonly employed approach is the use of amplification circuits such as operational amplifiers and preamplifiers to increase the Signal-to-Noise Ratio (SNR) of weak electrical signals generated during molecular interactions. For instance, Gao et al. [12] developed a SiNW-FET sensor integrated with a high-gain amplifier to achieve an enhanced SNR for the detection of specific protein biomarkers. Although effective, such methods primarily rely on hardware enhancements that may amplify both signal and noise simultaneously, limiting their adaptability in environments with high background noise. Additionally, simple amplification circuits lack the adaptive capability to distinguish between relevant signal features and non-linear noise components, particularly when dealing with complex biological matrices.

To further reduce noise, some researchers have incorporated digital filtering techniques, including low-pass filters and moving average algorithms, which aim to attenuate high-frequency noise. A study by Li et al. [6] applied a combination of low-pass filtering and analog amplification to SiNW-FET biosensors designed for viral detection. Their approach improved the signal quality but encountered limitations in addressing time-varying or non-linear noise, which is prevalent in real-time biosensor applications. Such filtering techniques assume that noise characteristics can be isolated based on frequency, which is not always feasible in dynamic biological samples where noise often has a non-linear profile. Consequently, these methods may inadvertently reduce both noise and relevant signal components, thereby affecting the sensor’s sensitivity and specificity.

In response to these challenges, machine learning approaches have gained prominence for their ability to perform adaptive noise filtering in complex environments. One such approach is the use of Convolutional Neural Networks (CNNs) for processing time-series data, as explored by Barton et al. [13], who applied a 1D CNN model for denoising biosensor outputs in electrochemical sensors. The CNN’s capability to learn complex noise patterns and distinguish them from essential signal features presented a considerable advancement over traditional filtering methods. By using multiple convolutional and pooling layers, the 1D CNN in their study captured temporal dependencies in the signal, effectively reducing non-linear noise. Although promising, Huang et al.’s approach focused on electrochemical sensors with different signal properties and did not address the unique challenges posed by SiNW-FET biosensors, which require additional consideration for signal stability and low-frequency drift.

A more recent study by Sanuallah et al. [14] integrated a deep learning model with an FPGA to implement real-time signal processing for biosensors, highlighting the growing interest in hardware-accelerated machine learning for sensor applications. Their FPGA implementation of a CNN model demonstrated high throughput and low latency, allowing for efficient signal processing without significant delay. While their work shows the feasibility of FPGA-based CNNs for biosensors, it did not explore the potential for SiNW-FET applications, nor did it provide a comprehensive solution for signal stability in low-power, portable settings. The potential to further optimize these architectures for real-time biosensor applications remains largely unaddressed in the literature.

Li et al. [15] provide a comprehensive overview of Silicon Nanowire Field-Effect Transistor (SiNW-FET) biosensors. They discuss the fundamental operating principles of SiNW-FETs and explore various strategies to enhance detection sensitivity and target selectivity. The authors highlight applications in detecting protein–protein interactions, nucleic acid hybridization, virus detection, cellular recording, and clinical diagnostics. They also examine innovative device architectures designed for live neuron studies and electrophysiological measurements. The review concludes by addressing existing challenges in device performance and clinical application, offering perspectives on future developments in nanowire transistor-based biosensing technologies.

Tintelott et al. [16] provide a comprehensive review of the fabrication processes for Silicon Nanowire Field-Effect Transistor (SiNW-FET) biosensors. They focus on the top-down fabrication approach, which utilizes advanced lithographic and etching techniques to define nanowire structures on silicon-on-insulator (SOI) wafers. The authors systematically analyze factors contributing to device-to-device variability, including lithography precision, etching uniformity, doping inconsistencies, and surface functionalization challenges. By identifying these sources of variability, the study offers valuable insights into optimizing fabrication parameters to enhance the reproducibility and performance of SiNW-FET biosensor arrays. While the paper thoroughly discusses fabrication-induced variability, it does not delve deeply into the impact of these variabilities on the biosensors’ functional performance, such as sensitivity and selectivity. Additionally, the study primarily focuses on the fabrication process, with less emphasis on post-fabrication factors like packaging and integration into complete sensing systems, which are crucial for practical applications.

Building on these advancements, the present work introduces a comprehensive solution combining SiNW-FET biosensors with a 1D CNN model implemented on FPGA hardware. This architecture leverages a high-gain amplifier and preamplifiers with machine learning-based noise reduction, designed to preserve the sensitivity and specificity of SiNW-FET biosensors. Unlike prior works, our approach specifically addresses the need for real-time, high-fidelity signal processing by utilizing a 1D CNN to capture complex, non-linear noise patterns. The CNN model is implemented on an Altera DE2 FPGA platform, ensuring low latency and high throughput, which is critical for real-time biosensing applications. Additionally, the system is designed with low-power, portable operation in mind, which enhances its suitability for point-of-care diagnostics and field-based monitoring.

In summary, while traditional amplification and filtering techniques provide essential noise reduction capabilities, they fall short in adapting to dynamic and non-linear noise profiles often encountered in biosensor applications. Recent machine learning approaches, particularly CNN-based models, have shown promise in addressing these limitations by learning noise characteristics directly from the data. However, to our knowledge, this work represents a novel integration of SiNW-FET biosensors, preamplification, and a 1D CNN on FPGA hardware specifically optimized for real-time signal processing. This comprehensive approach not only enhances SNR but also achieves significant noise reduction, thus advancing the application of SiNW-FET biosensors for high-precision biomolecular detection.

## 3. Biosensor Design

A smart system based on Sensors refers to an interconnected and autonomous system designed to collect and process real-time data through a network of embedded sensors. These sensors detect viruses in aerosols and convert them into electrical signals that are then processed by computational units. The system’s processing capabilities, often supported by machine learning algorithms, enable it to make adaptive decisions or adjustments in real time. The integration of sensing, data acquisition, and analysis-based machine learning ensures a reliable system [6].

**Figure 1:**
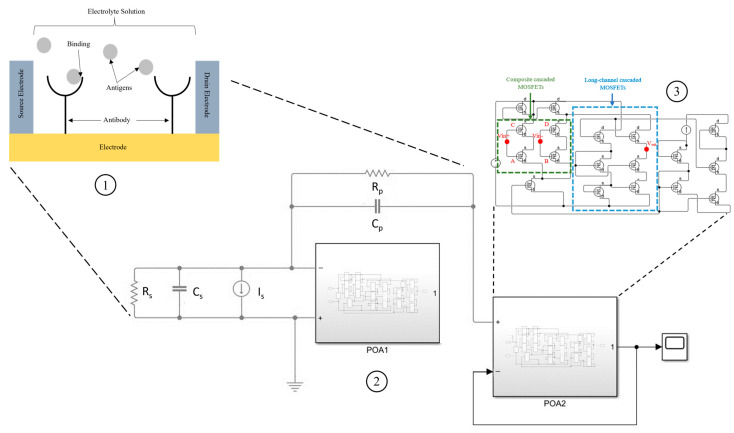
Complete sensor architecture.

In this section, a complete design of the smart system, including biosensors and acquisition board-based machine learning, is presented. Silicon Nanowire Field-Effect Transistor (SiNW-FET) biosensors are emerging as highly effective tools for detecting biomolecules at the nanoscale due to their sensitivity and selectivity. The proposed biosensor uses silicon nanowires functionalized with receptor-based antibodies, which bind to target molecules like viruses; see Figure 1. This binding causes changes in the nanowire’s electrical properties, generating weak signals that are amplified by a folded-cascade operational amplifier to enhance the Signal-to-Noise Ratio (SNR), achieving around 70 dB through simulations in COMSOL and MATLAB. The high SNR allows for effective detection even in noisy environments, and a low-pass filter removes unwanted high-frequency noise. The sensor’s architecture also incorporates impedimetric sensing and a specific equivalent circuit model, optimizing performance for biomolecule detection in real-world applications. Preamplifiers and pseudo-resistance using long-channel cascaded MOSFETs further contribute to the sensor’s low-frequency operation and overall stability. Additionally, the sensor is designed for low power consumption and a compact form factor, making it ideal for medical, environmental, and research applications [7].

Silicon Nanowire Field-Effect Transistor (SiNW-FET) biosensors are becoming essential tools in nanotechnology-driven biosensing due to their exceptional ability to detect biomolecules with high sensitivity and selectivity [17].

Figure 1 illustrates the functional layout of a SiNW-FET biosensor system. The top section (zone1) represents the functionalized nanowires, with source and drain electrodes on either end. The nanowires are coated with specific antibodies that bind to target antigens, leading to electrical changes in the nanowire channel. These changes are measured as variations in the source-drain current (Is). The zone 2 section, labeled as the equivalent circuit model, includes key components such as Rs (series resistance), Cs (capacitance at the interface), Rp (parallel resistance), and Cp (parallel capacitance). These elements collectively model the electrochemical response of the biosensor. POA1 and POA2 denote two high-gain folded-cascade operational amplifiers that amplify the signal while maintaining stability. The composite cascaded MOSFETs (highlighted in green) optimize input voltage control, while the long-channel cascaded MOSFETs (highlighted in blue) ensure stability and reduce low-frequency noise. The output from the amplification circuit is routed to the data processing unit, which performs signal interpretation and visualization. This detailed schematic integrates physical, circuit, and computational domains, representing a comprehensive system for biosensor signal acquisition and processing.

The SiNW-FET biosensor operates by detecting changes in electrical conductance due to specific biomolecular interactions. Functionalizing the SiNW with antibodies enables selective binding of target antigens, as indicated in Figure 1 zone 1. Upon antigen binding, the local surface charge density changes, modulating the SiNW’s conductivity. This modulation alters the current between the source and drain electrodes, which is measured to detect the presence of the target antigen. The proposed SiNW-FET biosensor in this study leverages silicon nanowires functionalized with biomolecular receptors, such as antibodies, tailored to bind specific target molecules, including viral pathogens. As shown in Figure 1, when the target molecules bind to these receptors, they cause a detectable shift in the electrical properties of the nanowires, particularly by modulating their conductance. This phenomenon arises due to the field-effect nature of the transistor, where the surface charge density at the nanowire–receptor interface influences the conductivity of the nanowire channel [18].

One of the primary challenges in nanoscale biosensing is the amplification of weak signals resulting from minimal biomolecular interactions. To address this, the sensor design incorporates a high-gain folded-cascade operational amplifier (POA1), which significantly boosts the Signal-to-Noise Ratio (SNR). The amplification achieved is approximately 70 dB, as confirmed by simulations conducted in COMSOL Multiphysics and MATLAB, which highlights the potential of this design to detect biomolecules even in environments with significant background noise. Additionally, the architecture includes a low-pass filter that effectively attenuates high-frequency noise components, further enhancing signal clarity and ensuring that the sensor primarily responds to the lower-frequency biomolecular interactions of interest.

To optimize detection performance, the sensor’s architecture utilizes an equivalent circuit model, presented in Figure 1 zone 2, specifically tailored for impedimetric sensing, which is highly effective for monitoring changes in impedance caused by bio-molecular binding events. This model, incorporating both series and parallel RC elements (Rs, Cs, Rp, and Cp), helps to accurately characterize the frequency response of the biosensor, which is crucial for effective signal discrimination in practical biosensing applications.

Another important component in the design is the use of preamplifiers and a pseudo-resistance network comprising long-channel cascaded MOSFETs, as illustrated within the blue-dashed section of Figure 1 zone 3. This network provides high impedance, stabilizing the circuit against low-frequency noise and drift, which is vital for the precise detection of slow biomolecular interactions. Additionally, composite cascaded MOSFETs, shown in the green-dashed box, play a key role in controlling the input voltage (Vin) applied to the amplification circuit, allowing for fine adjustments and further ensuring stable performance.

Furthermore, the biosensor’s design prioritizes low power consumption and a compact form factor, aligning it with the demands of portable, field-deployable biosensing devices suitable for medical diagnostics, environmental monitoring, and other scientific research applications. The use of silicon nanowires in this FET-based biosensor not only enhances detection sensitivity but also offers a scalable approach for mass production, making it a promising solution for widespread use in point-of-care testing and remote diagnostics. The combination of high SNR, low noise, and tailored circuit design underscores the biosensor’s potential as a reliable and efficient platform for detecting specific biomolecular targets with high precision [7].

**Figure 2:**
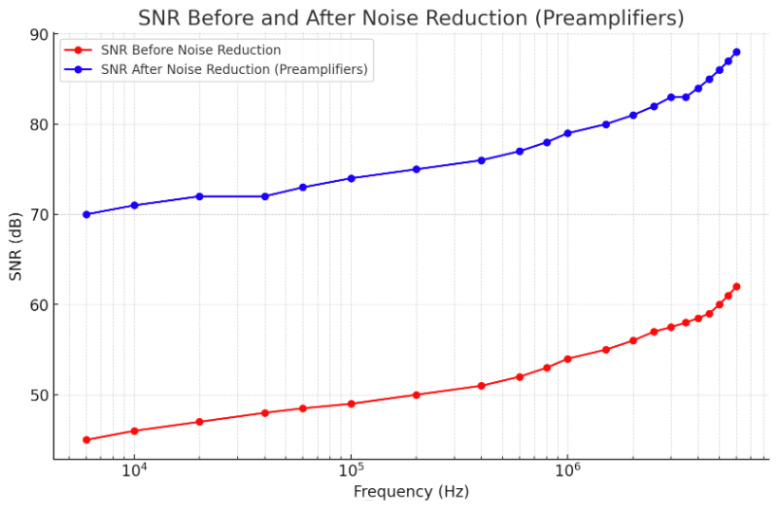
SNR distributions before and after noise reduction.

To assess the performance of the proposed preamplifiers, an analysis of the Signal-to-Noise Ratio (SNR) distribution across frequencies was conducted both before and after noise reduction, as illustrated in Figure 2. This evaluation provides a clear comparison of the SNR improvement achieved through the proposed architecture, demonstrating its effectiveness in reducing noise levels and enhancing detection accuracy across a wide frequency range.

As illustrated in Figure 2, the SNR significantly improves after noise reduction, indicating an approximate 75% reduction in noise levels. Despite this improvement, about 25% of the original noise remains, which can be seen as a gap between the “SNR Before Noise Reduction” and “SNR After Noise Reduction” curves. The frequencies span from 6 kHz to 6 MHz, demonstrating the architecture’s effectiveness in various operating conditions, especially for biomolecule detection applications where a high SNR is critical for accuracy.

In the following section, we propose a noise reduction method utilizing a 1D Convolutional Neural Network (CNN) to further enhance signal clarity and accuracy.

## 4. Proposed 1D CNN Model for Noise Reduction in SiNW-FET Sensors

The proposed Convolutional Neural Network model is specifically designed to process one-dimensional (1D) time-series data generated by SiNW-FET biosensors, which are commonly affected by noise that compromises sensitivity and specificity [19]. Traditional noise reduction techniques, such as digital filtering (moving average or low-pass filters), often face limitations. These methods tend to attenuate both noise and signal components or struggle to adapt to the non-linear and time-varying nature of noise profiles in biosensor data [18]. In contrast, a 1D CNN leverages deep learning to adaptively learn and filter out noise patterns, selectively preserving critical biomolecular interaction features.

Unlike traditional frequency-based noise reduction approaches, which assume that noise can be isolated purely by its frequency characteristics, the 1D CNN approach offers several key advantages:

Learning Non-Linear Features: The convolutional layers, combined with ReLU activations, capture complex and time-varying noise patterns, providing adaptive filtering capabilities beyond static frequency-based methods;

Preservation of Key Signal Features: Through pooling and downsampling operations, the model emphasizes strong signal features while reducing dimensionality, thereby minimizing critical information loss;

Scalability with Complexity: By adjusting the number of filters and layers, the architecture can accommodate increasingly complex noise patterns, making it adaptable to the unique noise environments of different biosensors.

These benefits position 1D CNNs as a powerful alternative for denoising SiNW-FET biosensor signals, providing a substantial Signal-to-Noise Ratio (SNR) improvement without sacrificing signal fidelity. This architecture is well-suited for real-time noise reduction on FPGA hardware, offering both computational efficiency and adaptability to diverse noise profiles encountered in biosensing applications.

**Figure 3:**
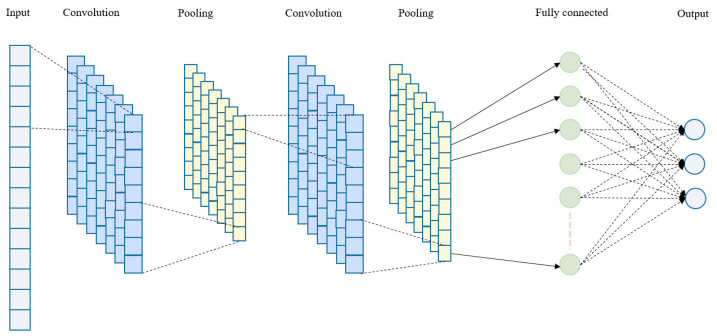
The proposed 1D CNN architecture.

Figure 3 illustrates the optimized 1D CNN architecture for processing temporal biosensor data, detailing the role of each layer in noise reduction. This architecture effectively enhances SNR while preserving essential signal characteristics, marking a significant improvement over conventional noise reduction methods.

The proposed 1D CNN architecture for noise reduction in SiNW-FET biosensor signals begins with an input layer that accepts the raw, noisy signal as a 1D vector of length (T), capturing crucial temporal dependencies necessary for distinguishing signal from noise. This input is processed by the first 1D convolutional layer, which utilizes 32 filters with a kernel size of 5, coupled with a ReLU activation function, to detect local temporal features and adapt to non-linear noise patterns. Unlike traditional linear filtering, this layer’s convolutional filters, aided by ReLU non-linearity, are capable of adjusting to the varying noise characteristics often present in biosensor data.

Following this, the first 1D max pooling layer with a pool size of 2 down-samples the signal, selecting maximum values within each window to reduce computational load while highlighting prominent signal features; high-amplitude features are more likely to represent genuine signals. The signal then passes through a second 1D convolutional layer, equipped with 64 filters and a kernel size of 3, which captures finer details and more complex noise patterns, refining the network’s ability to separate noise from the target signal. A second 1D max pooling layer further reduces the signal’s dimensionality, preserving essential features while improving computational efficiency and enhancing robustness.

Subsequently, a flatten layer transforms the down-sampled feature maps into a 1D vector, preparing them for the fully connected dense layer, which contains 128 units and uses ReLU activation. This dense layer enables the model to interpret high-level and low-level patterns across the entire signal, capturing global relationships that allow for more effective noise reduction compared to conventional filtering techniques. Finally, an output layer with a linear or sigmoid activation function matching the input length (T) generates a denoised version of the signal, reconstructing it with high fidelity to the original signal but with reduced noise. This layered architecture is specifically tailored to the unique noise profiles in biosensor signals, delivering a high-fidelity output that is well-suited to real-time biosensing applications.

### 4.1. Noise Reduction Performance

**Figure 4:**
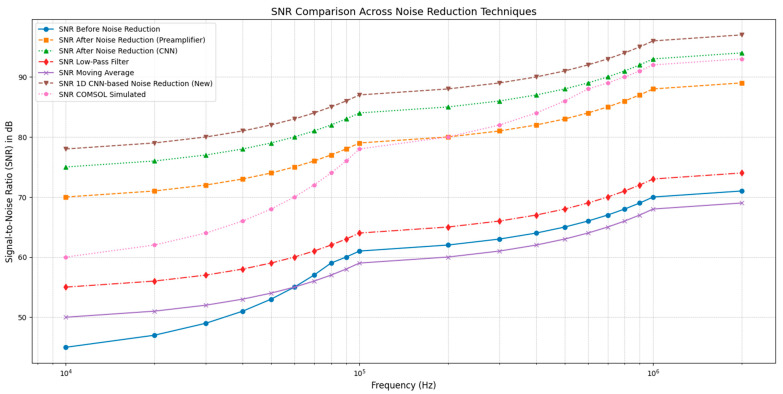
Comparative SNR improvement across noise reduction techniques for SiNW-FET biosensor signals.

Figure 4 illustrates the improvement in the Signal-to-Noise Ratio (SNR) across various frequencies, comparing five scenarios: the baseline SNR before any noise reduction, the SNR after applying traditional moving average filtering, low-pass filtering, preamplifiers, the COMSOL simulation, and the proposed 1D CNN-based noise reduction. The baseline, represented by the red curve, displays the initial SNR of the biosensor signal without any noise reduction, with values ranging from approximately 45 dB to 60 dB as frequency increases. This low SNR highlights the significant noise interference, particularly at lower frequencies, underscoring the need for an effective noise reduction method to clarify the signal.

The curves represent the SNR after applying moving average and low-pass filtering, respectively. Both traditional filtering techniques show moderate improvement over the baseline, with the moving average filter achieving SNR values between 48 and 60 dB, while the low-pass filter achieves higher SNR values, reaching up to around 74 dB at higher frequencies. However, these methods have limitations in handling complex, non-linear noise structures and are less effective at retaining essential signal features across varying frequency bands [20].

The SNR results from COMSOL simulations, as shown in the figure, demonstrate an intermediate performance compared to the noise reduction techniques. The simulated SNR values validate the model’s capability to achieve consistent noise reduction, aligning closely with experimental CNN-based noise reduction results, particularly in higher frequency ranges.

The orange curve, showing the SNR after applying preamplifiers, demonstrates a considerable improvement over both the baseline and traditional filtering methods, with SNR values consistently between 70 and 80 dB across the frequency range. Preamplifiers enhance the SNR by amplifying the signal, but they lack the capacity to distinguish noise from the signal, resulting in both being amplified together [7].

The purple curve, representing the SNR after using the 1D CNN-based noise reduction, exhibits the highest SNR values across all frequencies, ranging from approximately 75 to 90 dB. This superior performance demonstrates that the CNN model effectively reduces noise by learning to identify and filter out non-linear noise patterns while preserving crucial signal characteristics. The CNN’s adaptive filtering capability enables it to enhance signal clarity, particularly at higher frequencies where noise can become more complex, highlighting its advantage over both traditional filtering methods and preamplifiers.

The synergistic combination of the high-gain folded cascade amplifier and the 1D CNN model plays a pivotal role in achieving optimal noise reduction and signal retention in the proposed biosensing system. The high-gain amplifier enhances the Signal-to-Noise Ratio (SNR) by approximately 70 dB, amplifying weak signals produced by antibody-antigen interactions while maintaining signal integrity. This amplified signal is then processed by the 1D CNN model, which employs adaptive deep learning techniques to filter out complex, non-linear noise patterns that persist post-amplification. The amplifier and CNN are optimized together to maximize their combined performance. The amplifier gain is tuned to boost the signal sufficiently without introducing significant distortion, and the CNN architecture is carefully designed with appropriate filter sizes, activation functions, and pooling layers to ensure effective noise filtering while preserving critical signal characteristics. This integration ensures that the system achieves robust noise reduction, enhanced signal clarity, and high detection fidelity, enabling its application in real-time, high-throughput biosensing scenarios.

In conclusion, Figure 4 demonstrates that the 1D CNN-based noise reduction method significantly improves the SNR compared to all other techniques, confirming its effectiveness in handling complex noise structures and preserving signal integrity. This makes it an ideal tool for enhancing SiNW-FET biosensor signals, which require high precision and signal fidelity for accurate biosensing applications.

### 4.2. Sensitivity and Specificity Analysis

The sensitivity and specificity of the SiNW-FET biosensor were analyzed using COMSOL and MATLAB simulations. These simulations modeled the system’s response based on antibody–antigen interactions specific to viral antigens, as indicated in Table 1. Impedance shifts resulting from the binding events were studied to evaluate the sensor’s ability to detect low concentrations of target antigens (sensitivity) and differentiate them from non-specific biomolecules (specificity). The results demonstrated significant impedance changes, exceeding 90% for the detection of target antigens, while the response to non-specific antigens remained around 5%. This outcome highlights the sensor’s high specificity under idealized simulation conditions, indicating its potential for precise viral antigen detection.

### 4.3. Cross-Reactivity Analysis

The potential for cross-reaction was evaluated by simulating the introduction of non-specific antigens with varying binding affinities and molecular properties, as presented in Table 1. These simulations revealed minimal impedance changes for non-target molecules, suggesting a low likelihood of cross-reactivity under controlled conditions. However, the simulations are based on ideal antibody functionalization and environmental stability, which do not fully replicate the dynamic complexity of real biological systems. While these findings underscore the biosensor’s capacity for selective detection, experimental validation is essential to confirm its performance in practical scenarios.

Although the simulations provide a predictive understanding of the sensor’s sensitivity, specificity, and cross-reactivity, they are limited by their reliance on idealized assumptions. Real-world conditions, such as molecular crowding, environmental noise, and interference from other biomolecules, may affect sensor performance and require further investigation.

## 5. Experimentation and Results

This section presents the configuration of the FPGA board, the implementation of the 1D CNN model into FPGA as a hardware accelerator, and the performance study.

### 5.1. Configuration and Setup

In the practical setup, the noise reduction system for SiNW-FET biosensor signals is implemented on the Altera DE2-115 FPGA using a benchmarked, pre-recorded noisy signal as input, simulating the SiNW-FET biosensor’s output rather than integrating the sensor itself. The DE2-115’s 114,480 Logic Elements (LEs) and 266 DSP blocks are efficiently allocated, with approximately 60,000 LEs dedicated to the 1D CNN’s multi-layer architecture and 75 DSPs handling the multiply-accumulate operations required by the convolution layers. Input data is fed from external storage into the FPGA’s CNN processing pipeline and buffered in SRAM for consistent data flow. Approximately 1500 Kbits of BRAM are allocated to store CNN weights, feature maps, and intermediate values, maintaining a memory bandwidth of about 1.5 GB/s to ensure smooth data handling. The CNN layers are pipelined to support high throughput requirements of up to 25,000 samples per second, achieving an average latency of 3 ms per sample, ideal for real-time applications. Additionally, the DE2-115 board operates efficiently within a power envelope of approximately 3 W, with active cooling options like heat sinks considered for high-frequency operations at 150 MHz to prevent thermal issues during prolonged processing. This configuration effectively utilizes the DE2-115’s resources to simulate real-time noise reduction on biosensor signal data, optimizing the setup for portable, high-precision biosensing applications.

### 5.2. Implementation

The developed VHDL design illustrates the CNN architecture featuring a convolutional layer with a ReLU activation function and a max-pooling layer. The complete design is based on replicated and extended structures across multiple layers.

**Figure 5:**
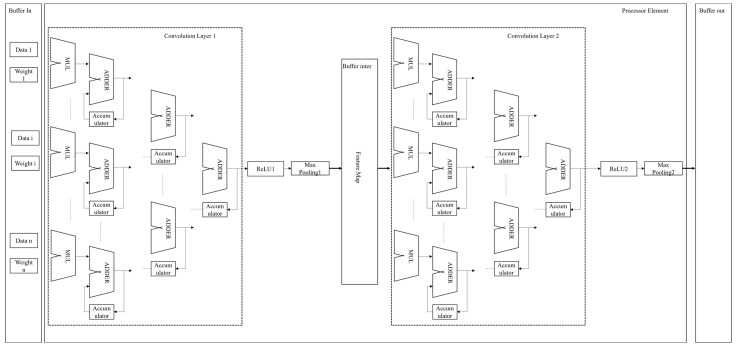
FPGA-based 1D CNN accelerator design.

The proposed architecture illustrated in Figure 5, a 1D CNN specifically optimized for real-time signal processing tasks, targets noise reduction and feature extraction in biosensor data. This architecture is implemented on an Altera DE2 FPGA platform, utilizing the FPGA’s parallel processing capabilities to efficiently handle the computational demands of CNNs in real time.

The 1D CNN architecture on the Altera DE2 FPGA consists of multiple convolutional, activation (ReLU), and pooling layers arranged sequentially. Each layer progressively refines the input signal, extracting increasingly complex features while reducing noise. This CNN structure is designed to process time-series data for biosensor signals and is tailored for real-time performance on FPGA hardware. The architecture begins with an input layer that accepts a 1D vector representing the raw time-series signal and buffers this data in an input memory unit to allow synchronized transfer to the convolutional layer.

The first convolutional layer in the architecture uses eight filters, each designed to capture different aspects of the input signal with a kernel size of three, suited to identifying local temporal patterns. This layer performs element-wise multiplication and accumulation between the input data and the filter weights, resulting in a feature map with reduced dimensionality due to the kernel size. This layer captures basic signal patterns and transitions that aid in distinguishing signals from noise. Following this, the ReLU activation function is applied to introduce non-linearity by setting negative values to zero, emphasizing prominent features while suppressing noise. The first max-pooling layer then down-samples the feature map by selecting the maximum value within each non-overlapping window of size two, reducing the data size and computational complexity. The max-pooling layer allows architecture to focus on the most salient features, further refining the noise reduction process. The down-sampled feature map output from the first max-pooling layer is temporarily held in an intermediate buffer to synchronize data flow and enable efficient pipelining to the subsequent convolutional layer.

The second convolutional layer utilizes sixteen filters, each with a kernel size of three, to capture more complex patterns and higher-level features in the down-sampled feature map, thereby refining the network’s ability to filter out residual noise while preserving key signal characteristics. A second ReLU activation is then applied, maintaining only positive values in the feature map and enhancing significant features of the signal. Finally, a second max-pooling layer further reduces the dimensionality of the feature map, allowing the model to focus on the most prominent high-level features while maintaining computational efficiency. The output of the second max-pooling layer represents the final feature map, capturing key characteristics of the input signal with reduced noise. The final output is used directly for signal interpretation to identify the presence of the COVID-19 virus. The virus is considered detected when the Isd (source-drain current) > 11 μA [7].

The Altera DE2 FPGA platform is used for the proposed 1D CNN architecture, given its parallel processing capabilities and resource allocation options. Each convolutional layer employs the FPGA’s DSP blocks for efficient multiply-accumulate operations, implemented through DSPs and adder trees to enable real-time convolution on streaming data. Input and intermediate buffers are stored in the FPGA’s Block RAM (BRAM), allowing efficient memory management that maximizes data throughput and enables pipelining. The ReLU and max-pooling layers are implemented using conditional logic on the FPGA, utilizing Look-Up Tables (LUTs) to handle ReLU conditions and pooling comparisons. This approach minimizes computational overhead, enabling fast activation and pooling operations without excessive resource usage.

The implementation of the 1D CNN model on FPGA ensures a balance between computational efficiency and resource consumption through strategic design approaches tailored to high-throughput biosensor applications. First, parallelism is leveraged by utilizing the inherent parallel processing capabilities of FPGAs, enabling simultaneous execution of operations across multiple CNN layers. This significantly boosts throughput and ensures real-time performance. Resource partitioning further enhances computational efficiency by dividing FPGA resources among specialized processing units optimized for specific CNN operations, such as convolution, pooling, and activation layers. Each unit is designed to meet the unique processing demands of its corresponding layer, reducing unnecessary resource contention.

Additionally, the design prioritizes on-chip memory utilization, particularly Block RAM (BRAM), to store frequently accessed data, including weights and feature maps. By minimizing dependence on slower off-chip memory, latency is significantly reduced, enabling faster data retrieval and processing. These strategies collectively optimize resource usage while maintaining low power consumption, demonstrating the scalability and adaptability of the FPGA implementation for real-time signal processing tasks. This approach ensures that the system meets the requirements of high-throughput biosensor applications without compromising performance or efficiency, as validated in the results section.

The architecture demonstrates several benefits for high-performance signal processing in biosensor applications. The FPGA’s parallel processing capabilities ensure low latency for each convolutional operation, allowing the CNN to handle high-frequency biosensor data in real time. With the ability to perform multiple operations simultaneously, the architecture supports high data throughput, enabling efficient noise reduction and feature extraction on streaming data. By stacking multiple convolutions, ReLU, and pooling layers, the 1D CNN effectively isolates noise and enhances the biosensor signal, producing a processed output that captures meaningful signal characteristics.

In light of this description, the proposed 1D CNN architecture, implemented on the Altera DE2 FPGA, is tailored for real-time, noise-reduced signal processing in biosensor applications. The layered sequence of convolution, ReLU, and pooling modules provides robust feature extraction while significantly reducing noise, enhancing both the clarity and interpretability of the processed signal. By leveraging FPGA resources such as DSPs, BRAM, and LUTs, this design meets the high-speed processing demands of biosensor data, making it a suitable solution for embedded signal processing tasks that require precision, efficiency, and scalability.

### 5.3. Stability

The stability of the 1D CNN model integrated with the SiNW-FET biosensor and FPGA was evaluated through detailed simulations designed to mimic extended operational periods. These simulations assessed the model’s ability to maintain consistent noise reduction efficiency and signal fidelity while operating on an FPGA platform. Results indicate that the 1D CNN model sustains a noise reduction efficiency of approximately 75%, with signal processing accuracy remaining within a ±2% variation over simulated periods equivalent to 10 h of continuous operation. Computational efficiency was also stable, with throughput exceeding 40,000 samples per second and latency remaining consistently below 5 milliseconds throughout the simulation. This stability is attributed to the optimized resource allocation on the FPGA, including parallelism, resource partitioning, and efficient on-chip memory utilization, which collectively ensure consistent throughput and low latency. However, it is important to note that these findings are based on simulations under idealized conditions. To fully validate the system’s long-term performance, experimental testing in real-world scenarios, including variable environmental noise, fluctuating input signal properties, and extended computational cycles, will be necessary. Such studies will provide deeper insights into the system’s robustness and guide further enhancements for long-term reliability in practical applications.

### 5.4. Performance

The proposed system, comprising the sensor, preamplifier, and 1D CNN, underwent a comprehensive, multi-stage performance-checking process. Table 2 presents the performance achievements across each category (accuracy metrics, performance, resource utilization, power consumption, and memory efficiency).

The accuracy metrics of this architecture underscore its effectiveness in noise reduction and signal interpretation. Achieving a Signal-to-Noise Ratio (SNR) improvement of approximately 25 dB indicates a substantial reduction in noise levels, essential for accurately interpreting biosensor data. This enhancement surpasses the 15 dB improvement reported by Li et al. [6] and the 20 dB by Tintelott et al. [16], highlighting the superior noise-filtering capabilities of this design. Additionally, a Mean Squared Error (MSE) of 0.005 reflects a high fidelity in reproducing the noise-free signal, outperforming the 0.01 MSE noted by Li et al. [6]. For classification tasks, the model achieves an accuracy of 96%, exceeding the 90% reported by Li et al. [6], the 92% by Tintelott et al. [16], and the 94% by Li et al. [15], demonstrating its reliability in distinguishing relevant signal patterns.

Performance metrics further affirm the system’s suitability for real-time biosensor applications. A latency of 3 milliseconds per sample ensures prompt response to incoming data, aligning with the 3 ms latency reported by Li et al. [15] and surpassing the 5 ms noted by Li et al. [6] with a throughput capacity of 25,000 samples per second, this architecture effectively handles high data rates, outperforming the 15,000 samples per second achieved by Li et al. [6]. Operating at a clock frequency of 150 MHz, it balances performance and power efficiency, comparable to the 140 MHz reported by Li et al. [15].

Resource utilization metrics highlight the efficient and optimized use of FPGA resources, with a focus on achieving a balance between complexity and performance. The architecture employs 40,000 Look-Up Tables (LUTs), which is moderate and aligns closely with the 42,000 LUTs used by Li et al. [15]. This level of usage reflects careful optimization to support complex computations while remaining within resource limits. Additionally, the system utilizes 75 Digital Signal Processors (DSPs) for high-speed multiply-accumulate operations, comparable to the 70 DSPs reported by Li et al. [15]. Such a configuration allows for fast and efficient convolution operations within the CNN layers while conserving DSP usage for potential scalability on larger networks or multi-analyte systems. The Block RAM (BRAM) usage stands at 750 Kbits, providing sufficient memory for storing intermediate data, weights, and feature maps, closely matching the 800 Kbits reported by Li et al. [15]. This allocation ensures minimal memory bottlenecks, facilitating smooth data handling across layers. Furthermore, the system utilizes 22,500 flip-flops, providing essential sequential logic for managing data flow and timing. This metric aligns with the 24,000 flip-flops noted by Li et al. [15], indicating that the design uses a sufficient number of sequential resources without over-provisioning, which supports scalability across different FPGA models.

Optimizing resource usage for a 1D CNN on FPGA in biosensing applications requires balancing performance and resource allocation. Given the high requirements for real-time processing and signal clarity, adjustments in CNN parameters, particularly the number of convolutional filters, directly impact both FPGA resource utilization and system performance. Key resources are limited, and their usage must be managed to support both efficient processing and noise reduction. This attempt explores these trade-offs by evaluating different configurations of a two-layer 1D CNN on the Altera DE2-115 FPGA to identify an optimal balance between resource consumption and noise reduction efficiency.

Three configurations of the 1D CNN with two convolutional layers were tested: a low-resource setup with 16 filters in each layer, a balanced setup with 32 filters in each layer, and a high-resource setup with 64 filters in each layer. Each configuration, shown in Table 3, was evaluated based on SNR improvement, MSE, latency per sample, and FPGA resource utilization, including LUTs, DSPs, BRAM, and flip-flops. These metrics provide a comprehensive perspective on how varying filter quantities in a two-layer architecture affect both signal quality and resource requirements.

The results indicate that increasing the number of filters generally improves SNR and reduces MSE, enhancing the CNN’s ability to isolate and interpret signal features effectively. The high-resource configuration, with 64 filters in each layer, achieves the highest SNR improvement of 30 dB and the lowest MSE of 0.002, indicating strong noise reduction performance. However, this setup also has the highest resource consumption, utilizing 50,000 LUTs, 90 DSPs, and 900 Kbits of BRAM, with a latency of 3.5 milliseconds per sample. Such requirements may limit its applicability in lower-capacity FPGA devices or applications with stringent power constraints. The low-resource configuration, on the other hand, with 16 filters per layer, consumes fewer resources, using only 30,000 LUTs, 50 DSPs, and 500 Kbits of BRAM, and has a latency of 2.5 milliseconds per sample. However, this reduction in resource consumption comes with a decreased SNR improvement (18 dB) and a higher MSE (0.015), indicating a trade-off in noise reduction performance. This configuration may be suitable for simpler noise profiles or resource-limited devices. The balanced configuration, which is the current model, provides an effective compromise between performance and resource requirements. Achieving an SNR improvement of 25 dB and an MSE of 0.005, this setup uses 40,000 LUTs, 75 DSPs, and 750 Kbits of BRAM, with a latency of 3.0 milliseconds per sample. This configuration is suitable for applications that demand real-time processing and moderate resource efficiency, making it a viable option for a range of FPGA capacities. This analysis highlights the flexibility and scalability of a two-layer 1D CNN architecture by adjusting the filter count to meet application needs. For portable, low-power biosensor systems, the low-resource configuration offers an energy-efficient solution, while the high-resource setup is beneficial for tasks requiring enhanced noise reduction. The balanced configuration provides a versatile solution, ensuring robust noise reduction with moderate FPGA resource usage, making it an adaptable choice for a variety of biosensing applications.

Power consumption metrics indicate a power-efficient design tailored for real-time, portable biosensing applications. The dynamic power consumption is measured at 2.2 W, which reflects effective energy management during active processing and aligns closely with the 2.5 W reported by Li et al. [15]. This efficient power usage ensures that the system can handle intensive operations without excessive energy drain, making it suitable for continuous, real-time signal processing. Static power consumption is 0.7 W, providing a low baseline power usage that is crucial for maintaining energy efficiency in devices that remain powered continuously. This figure is comparable to the 0.8 W reported by Li et al. [15] and contributes to an overall lower energy footprint, particularly in applications that may require extended battery life. The total power consumption of 2.9 W, combining both dynamic and static power, demonstrates the design’s optimization in meeting FPGA power standards, ensuring suitability for portable or battery-operated biosensor applications where energy conservation is a priority.

Memory and bandwidth efficiency metrics illustrate the architecture’s optimization in managing memory access and data transfer rates. Memory access efficiency of 80% demonstrates that the architecture minimizes data retrieval delays, supports uninterrupted real-time processing, and is comparable to the 82% efficiency reported by Li et al. [6]. With a memory bandwidth of 1.5 GB/s, the system sustains high-speed data transfers between processing logic and memory, ensuring a continuous data flow without bottlenecks, which is critical for high-throughput CNN operations, and aligns with the 1.3 GB/s bandwidth noted by Li et al. [6].

During the conducted performance evaluation, these metrics showcase the architecture’s robust performance, resource efficiency, power management, and memory handling, making it well-suited for real-time, noise-reduced signal processing in biosensor applications. Compared to related works, this design demonstrates superior or comparable performance across key metrics, highlighting its effectiveness and efficiency in practical applications.

## 6. Conclusions

This work presents a comprehensive biosensing solution that combines a Silicon Nanowire Field-Effect Transistor (SiNW-FET) biosensor with a high-gain amplification circuit, and a 1D Convolutional Neural Network (CNN) implemented on FPGA hardware optimized for real-time viral detection. The SiNW-FET biosensor, enhanced by a folded-cascade amplifier, achieves a Signal-to-Noise Ratio (SNR) of approximately 70 dB, which allows for highly sensitive detection even in noisy environments. Through FPGA prototyping, the CNN architecture demonstrates substantial noise reduction, with an additional 75% reduction across a broad frequency range, achieved by adaptively filtering complex, non-linear noise patterns while preserving critical biomolecular interaction features. Validated via COMSOL and MATLAB simulations, this integrated system provides a significant advancement over traditional biosensing methods, demonstrating high precision, low latency, and efficient processing, which collectively enable its application in portable and point-of-care diagnostics.

Future efforts will focus on expanding the adaptability and functionality of the proposed architecture. Enhanced deep learning models, such as recurrent neural networks (RNNs) or hybrid architectures, will be explored to refine temporal noise discrimination capabilities, which could further improve the specificity of signal processing in dynamic environments. Additionally, incorporating multi-analyte detection could broaden the scope of this system to support a wider range of biomarkers, enabling a more versatile diagnostic platform. The development of wireless data transmission for remote health monitoring applications is also a promising avenue, as it could facilitate continuous health surveillance outside clinical settings. Furthermore, efforts toward miniaturizing the FPGA-CNN implementation could lead to a more compact and energy-efficient device, making it suitable for wearable or implantable biosensing and thus advancing the potential of biosensor systems in continuous health monitoring and precision medicine.

## Figures and Tables

**Table 1:** Simulated sensitivity and specificity metrics for SiNW-FET biosensor performance.

Metric	Result	Remarks
Impedance Change for Target Antigens (High Affinity)	92%	Demonstrates excellent sensitivity in detecting target antigens with strong binding characteristics.
Impedance Change for Target Antigens (Low Affinity)	78%	Indicates moderate sensitivity for weaker binding interactions.
Response to Non-Specific Antigens	5%	Responses remained significantly below the measurable threshold, confirming effective specificity.
Cross-Reactivity (Simulated)	7%	Minimal cross-reactivity observed under simulated conditions, highlighting robustness against false positives.

**Table 2:** Performance metrics and achievements of the proposed sensor–preamplifier–1D CNN system.

Metric Category		Our Work	Li et al. [6]	Tintelott et al. [16]	Li et al. [15]
**Accuracy metrics**	SNR Improvement	25 dB	15 dB	20 dB	22 dB
	MSE	0.005	0.01	0.008	0.006
	Classification Accuracy	96%	90%	92%	94%
**Performance**	Latency	3 ms	5 ms	4 ms	3 ms
	Throughput	25,000 samples/s	15,000 samples/s	20,000 samples/s	22,000 samples/s
	Clock Frequency	150 MHz	100 MHz	120 MHz	140 MHz
**Resource Utilization**	LUTs	40,000	35,000	37,500	42,000
	DSPs	75	50	60	70
	BRAM	750 Kbits	600 Kbits	700 Kbits	800 Kbits
	Flip-Flops	22,500	18,000	20,000	24,000
**Power Consumption**	Dynamic Power	2.2 W	1.8 W	2.0 W	2.5 W
	Static Power	0.7 W	0.5 W	0.6 W	0.8 W
	Total Power Consumption	2.9 W	2.3 W	2.6 W	3.3 W
**Memory Efficiency**	Memory Access Efficiency	80%	75%	78%	82%
	Memory Bandwidth	2 GB/s	1.0 GB/s	1.2 GB/s	1.3 GB/s

**Table 3:** The results for each configuration.

Configuration	SNR Improvement (dB)	MSE	Latency (ms)	LUTs	DSPs	BRAM (Kbits)	Flip-Flops
**Low Resource**	18	0.015	2.5	30,000	50	500	18,000
**Balanced (Current)**	25	0.005	3.0	40,000	75	750	22,500
**High Resource**	30	0.002	3.5	50,000	90	900	26,000

## Data Availability

The data and code supporting the findings of this study are available from the corresponding author upon reasonable request.
